# A student trained convolutional neural network competing with a commercial AI software and experts in organ at risk segmentation

**DOI:** 10.1038/s41598-024-76288-y

**Published:** 2024-10-29

**Authors:** Sophia L. Bürkle, Dejan Kuhn, Tobias Fechter, Gianluca Radicioni, Nanna Hartong, Martin T. Freitag, Xuefeng Qiu, Efstratios Karagiannis, Anca-Ligia Grosu, Dimos Baltas, Constantinos Zamboglou, Simon K. B. Spohn

**Affiliations:** 1 Department of Radiation Oncology, University Medical Center Freiburg, Faculty of Medicine, University of Freiburg, Freiburg, Germany; 2 Division of Medical Physics, Department of Radiation Oncology, University Medical Center Freiburg, Faculty of Medicine, University of Freiburg, Freiburg, Germany; 3 German Cancer Consortium (DKTK), Partner Site Freiburg, Freiburg, Germany; 4 Department of Nuclear Medicine, University Medical Center Freiburg, Faculty of Medicine, University of Freiburg, Freiburg, Germany; 5 Department of Urology, Affiliated Drum Tower Hospital, Medical School of Nanjing University, Nanjing, China; 6 German Oncology Center (GOC), European University of Cyprus, Limassol, Cyprus; 7 Berta-Ottenstein-Programme, Faculty of Medicine, University of Freiburg, Freiburg, Germany

**Keywords:** Prostate cancer, Radiation treatment planning, Auto segmentation, Convolutional neural network, Artificial intelligence, Turing test, Prostate cancer, Imaging techniques, Machine learning, Cancer imaging

## Abstract

This retrospective, multi-centered study aimed to improve high-quality radiation treatment (RT) planning workflows by training and testing a Convolutional Neural Network (CNN) to perform auto segmentations of organs at risk (OAR) for prostate cancer (PCa) patients, specifically the bladder and rectum. The objective of this project was to develop a clinically applicable and robust artificial intelligence (AI) system to assist radiation oncologists in OAR segmentation. The CNN was trained using manual contours in CT-datasets from diagnostic ^68^Ga-PSMA-PET/CTs by a student, then validated (n = 30, PET/CTs) and tested (n = 16, planning CTs). Further segmentations were generated by a commercial artificial intelligence (cAI) software. The ground truth were manual contours from expert radiation oncologists. The performance was evaluated using the Dice-Sørensen Coefficient (DSC), visual analysis and a Turing test. The CNN yielded excellent results in both cohorts and OARs with a DSC_median_ > 0.87, the cAI resulted in a DSC > 0.78. In the visual assessment, 67% (bladder) and 75% (rectum) of the segmentations were rated as acceptable for treatment planning. With a misclassification rate of 45.5% (bladder) and 51.1% (rectum), the CNN passed the Turing test. The metrics, visual assessment and the Turing test confirmed the clinical applicability and therefore the support in clinical routine.

**Supplementary Information:**

The online version contains supplementary material available at 10.1038/s41598-024-76288-y.

## Introduction

Over the past few years, artificial intelligence (AI) has revolutionized medical technology and research, and is increasingly being implemented in clinical practice. With the aim of not only facilitating and improving work processes, but also extending and precisionizing diagnostic and therapeutic options, AI has already become an indispensable part of the medical field, particularly in radiation oncology^1,2^. Radiotherapy (RT) is one of the main curative options for the most common malignant tumor diagnosed in men: prostate cancer (PCa). Technological advances allow precise application of high doses while sparing organs at risk (OAR) such as the bladder and rectum. To enable a high-quality radiation treatment, it is important to optimize and reevaluate workflow processes. Due to an increasing number of patients with a PCa, continued high-quality working routines are needed. For this purpose, automized processes supported by AI can be an important tool to support radiation oncologists in delineation of OARs, while OAR segmentation is mostly done manually, which is time consuming. Nowadays, RT can be already supported by licensed and costly AI software (commercial Artificial Intelligence, cAI).

The aim of the study was to train a freely available AI specifically for OAR segmentation in radiation treatment planning of PCa. One type of AI is Artificial Neural Networks (ANN), which consist of connected neurons inspired by biological neural nets. These ANNs are used and adapted in many fields of computer science to solve problems of various kinds. Convolutional Neural Networks (CNN) are a special type of ANNs, that are commonly used to accomplish computer vision tasks. CNNs for automatic organ segmentation are part of various analysis for multiple tumor entities and have delivered promising results^3,4^.

The CNN chosen for this project is a self-adaptive network first published by Isensee et al.^5^ and can be trained for different types of organ segmentation tasks. Other AI software, such as the web-based Total Segmentator^6^ has already been trained to automatically generate organ structures. However, this software can segment the bladder and the colon, but not the rectum, which is why Total Segmentator did not qualify for this project.

## Methods

First, the CNN was trained using multi-center computer tomography (CT) datasets only from positron emission tomography/CT (PET/CT) combined with tracers targeting the prostate-specific membrane antigen (PSMA). In a second step, the CNN was applied to an unknown validation cohort with PET/CT datasets and a test cohort with planning CT-datasets. Finally, the resulting CNN-segmentations as well as provided commercial Artificial Intelligence (cAI) segmentations were compared to those of clinical experts, set as ground truth, by calculating the Dice-Sørensen Coefficient (DSC), visual analysis and a Turing test to evaluate the clinical applicability.

### Preprocessing

#### Patients and datasets

This multi-center study, with datasets from three sites (center 1: Freiburg, Germany; center 2: Nanjing, China; center 3: Limassol, Cyprus), included patients with primary PCa and available imaging datasets. The local ethics committee approved the study (Ethics committee University of Freiburg Medical Centre, vote 469/14; 21-1149). Due to the retrospective nature of the study, the ethics committee of the University of Freiburg Medical Centre waived the need of obtaining informed consent. All methods described were conducted in accordance with the ethical principles outlined in the Declaration of Helsinki.

Exclusion criteria was a prior treatment such as prostatectomy or transurethral prostatic resection (TURP or laser assisted HOLEP). In the following, image dimensions and voxel spacing are provided for the x (right-left), y (anterior–posterior), and z (cranio-caudal) directions. At site 1, PET/CT-scans were acquired with a GEMINI TF 16 Big Bore Scanner (Philips Medical Systems) with a voxel spacing of 1.0x1.0x3.0 mm and image dimension of 512x512 with a median z of 312. At site 2, scans were done with an uMI 780 PET/CT scanner (United Imaging Healthcare) with a voxel spacing of 1.1x1.1x1.5 mm and image dimension of 512x 512 and a median z of 501. Both used ^68^Ga-PSMA as the radiopharmaceutical. The screening period was from April 2014 until April 2019. All DICOM datasets were anonymized, locally stored and pseudonymized. The datasets included native images, scans with intravenous as well as oral combined with intravenous contrast agents.

**Fig. 1:**
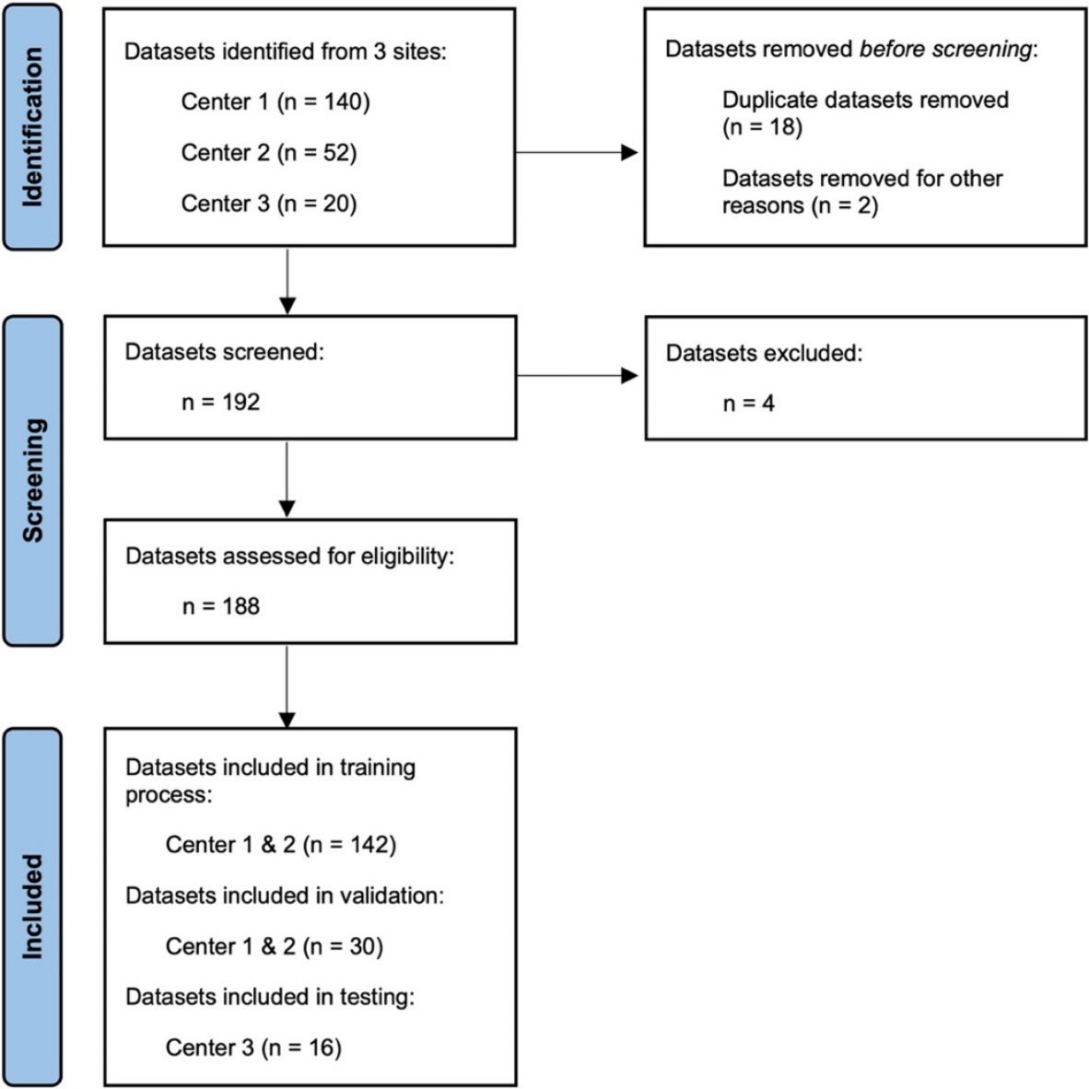
PRISMA workflow of the identification and screening process. In addition to the screened and excluded data, the randomization into the different cohorts can be seen in the included data.

For further analysis, 20 planning CT series from site 3, acquired with an Optima CT580 scanner (GE Medical Systems) during the period from January through August 2020, were provided. Voxel spacing was 0.9x0.9x2.5 mm and an image dimension of 512x512 with a z depending on scan length of 144 to 191. Datasets from site 3 were sent anonymized, stored locally and checked for exclusion criteria. Due to large resection cavities after transurethral resection of the prostate, four patients of site 3 had to be excluded. The screening process is shown in a PRISMA workflow (Fig. 1).

In total, 188 patients were included for further processing. Table 1 shows the distribution to the cohorts (further information such as patient specific characteristics and TNM-status for each center can be found in the Supplementary, Table S1.). For this project, 142 patients from centers 1 and 2 were selected as a training cohort, while 30 patients were randomized as a validation cohort for initial testing after completion of the training process. Patients from center 3 were designated as the test cohort for further analysis.

**Table 1:** Patient distribution. This table displays the cohort distribution to the training, validation and test cohort.

	Center 1: Freiburg, Germany	Center 2: Nanjing, China	Center 3: Limassol, Cyprus
n _total_	122	50	16
n _training_	99	43	0
n _validation_	23	7	0
n _test_	0	0	16

In a final step of preprocessing, the images of all datasets (training cohort, n = 142) were resampled to a uniform voxelspacing of 2 mm in each direction with BSpline interpolation. Contours were resampled accordingly with nearest neighbor interpolation. Afterwards CT and contour files were converted to nearly raw raster data (nrrd) format.

#### Segmentation of organs at risk and primary check

In this project, segmentations of both OARs bladder and rectum were assessed. The contouring followed the guidelines of the European Society for Radiotherapy and Oncology (ESTRO)^7^. The bladder was contoured with its complete craniocaudal extension. The segmentations of the rectum started aboral to the sigmorectal flexure. In air-filled sections, the hyperdense delineated rectal wall was included. For all segmentation processes, ‘3D Slicer’ program (version 4.11)^8^ was used and window levels were set to -135 to 215 Hounsfield units.

The segmentations for the training process have been contoured by a medical student after training under the supervision of two experienced radiation oncologists. Prior to the delineation, an initial assessment and evaluation of the medical student’s contouring abilities was conducted. The anatomical and fundamental radiological knowledge served as the foundation for this part. Following an introduction to the ‘3D Slicer’, the first segmentations were delineated. To improve and discuss any issues, a visual layer-by-layer analysis of the first five contours was conducted by the student and one expert involved in this project.

In a subsequent step, a primary check of the student’s skills was assessed prior to the upcoming training process and to prevent a systematic bias, Therefore, 30 random student-segmentations from center 1 were compared to the segmentations of the board-certified expert and analyzed by calculating the DSC using Python 3 library MedPy (version 0.4.0). The training process was continued after reaching a DSC threshold of > 0.6 predefined in the study design by the project managers for this primary check.

Then, the remaining segmentations of the training cohort were manually created by the student on the provided CT-datasets and saved as nrrd files.

### CNN and training process

The applied CNN by Isensee et al.^5^ is currently one of the most advanced CNNs for automatic segmentation of medical images. It is a self-configuring network with predefined parameters for different image modalities that automatically adapts to new segmentation tasks. Regarding the used U-shaped architecture, the trained CNN is called a ‘nnU-net’, which are well known for medical segmentation tasks.

For this study, the 3D full-resolution version of the nnU-Net was utilized. The 3D CT images (training cohort, n = 142) were used as input, resulting in three 3D output images, with the channels defined as follows: 0 for background, 1 for bladder, and 2 for rectum. The fixed predefined training parameters from the original study by Isensee et al. were used for processing. Hyperparameters like the depth of the CNN, number of filters, selected normalization methods and cost function are automatically defined by the nnU-Net, and hence are not further discussed in this paper (see Isensee et al.^5^ for details). The entire training process was carried out by a computer science student, with advice and verification from an experienced computer scientist.

### Preparation of comparative segmentations, validation and testing

After completing the training process, the trained CNN was utilized to generate the OAR segmentations of the unknown validation cohort (n = 30) datasets from centers 1 and 2. The resulting segmentations were saved as nrrd-files. To assess the CNN’s performance and resulting segmentations, two experienced and board-certified radiation oncologists (expert 1 and 2), specialized in urogenital cancer, manually created additional OAR segmentations of the validation cohort.

For testing, the datasets of center 3 (n = 16) with its planning CTs was delineated by another experienced radiation oncologist, named ‘expert 3’. All segmentations were approved by the board licensed expert before conducting the analysis. To avoid bias in organ segmentation, there was no prior review of the CNN-generated structures.

For further analysis, a commercial artificial intelligence (cAI) created OAR segmentations of both cohorts. Therefore, an auto contouring software by Limbus Contour (Version 1.5.0, Limbus AI Inc., Regina, SK, Canada) was used. Limbus Contour is a global auto contouring software that can be used for multiple tumor entities undergoing radiotherapy, including brain tumors, head and neck cancer, thoracic, gastrointestinal and urogenital tumors. It provides various organ contours and is a validated Artificial Intelligence that is often used in RT planning.

### Analysis

#### Metrics

For statistical analysis the segmentations were analyzed and the DSC was calculated with MedPy (Version 0.4.0). The CNN- and cAI-generated segmentations were respectively compared to the expert segmentations, which were considered as gold standard.

#### Qualitative characteristics

##### Visual analysis

Following a paper by Sherer et al.^9^ on visual assessment of AI segmentations, all CNN structure sets were assessed by two experienced radiation oncologists who were not involved in contouring. For this reason, the segmentations were uniformly visualized in ‘3D Slicer’ and classified by the investigators into one of three categories to objectify the clinical usability: Structures approved by the investigators concerning an implementation in a radiation treatment plan were assigned to group (1) ‘Accepted’. If minor corrections were required e.g. due to deviations from organ boundaries, structures were assigned to group (2) ‘Minor Deviation’ and if major aberrations were identified, structures were assigned to group (3) ‘Major Deviation’.

##### Turing test

When evaluating automatically segmented structures, the discrimination between a manually contoured and machine-generated origin is often questioned^10^, and therefore a Turing Test was designed. Four independent observers with different expertise (> 2, > 3, > 4, > 8 years), were asked to classify anonymized segmentations under the aspect of the origin. For this purpose, OAR structures of both cohorts were randomly selected from the expert and CNN structure sets and presented to the observers. The distribution in both cohorts was 1:1. After the assessment, the correct and false answers were detected. Then, the misclassification rate for each observer, each origin and each OAR were calculated using the formula: *misclassification rate* = *misclassifications/total assignments.* In line with literature by Ouyang et al.^11^, a threshold of 30% was set prior to the analysis. If the total misclassification for an OAR is below 30%, the segmentations can be distinguished from each other and dedicated to their origin. If the misclassification rate is higher, this student-trained CNN passes the Turing test.

## Results

### Metrics

Ahead of the training process, the manually created reference segmentations were compared to the expert segmentations as the primary check and showed a median DSC_Bladder_ = 0.92 (0.89–0.93) and DSC_Rectum_ = 0.87 (0.86–0.88). After exceeding the cut-off DSC > 0.6, the project was continued.

The CNN training, a first validation and post-processing were completed within 60 days, and the automatic generation of the segmentations was finalized. The mean processing for the CNN was 29 min and 29.4 s per patient, utilizing an Intel® Xeon® Gold5218R CPU @2.10 GHz (2 processors), 96.0 GB RAM and Quadro RTX 6000 GPU. The cAI created segmentations with a mean computing time of 68.0 s using a Common KVM processor @2.20 GHz (2 processors) and 16.0 GB RAM. With the exception of one dataset, in which case the segmentation could not be successfully generated due to poor image quality with coarse pixels, all cAI datasets were processed. To provide a fair comparison, this patient was excluded in the analysis (validation cohort: n = 29).

The comparison of the validation cohort between the CNN-segmentations and those of the experts 1 and 2 (I) resulted in a median DSC_Bladder_ = 0.93 and DSC_Rectum_ = 0.87. The cAI-segmentations compared to the expert segmentations (II) showed a median DSC_Bladder_ = 0.90 and DSC_Rectum_ = 0.78, with two rectum segmentations below the threshold of 0.6 (DSC_min_ = 0.39).

**Fig. 2:**
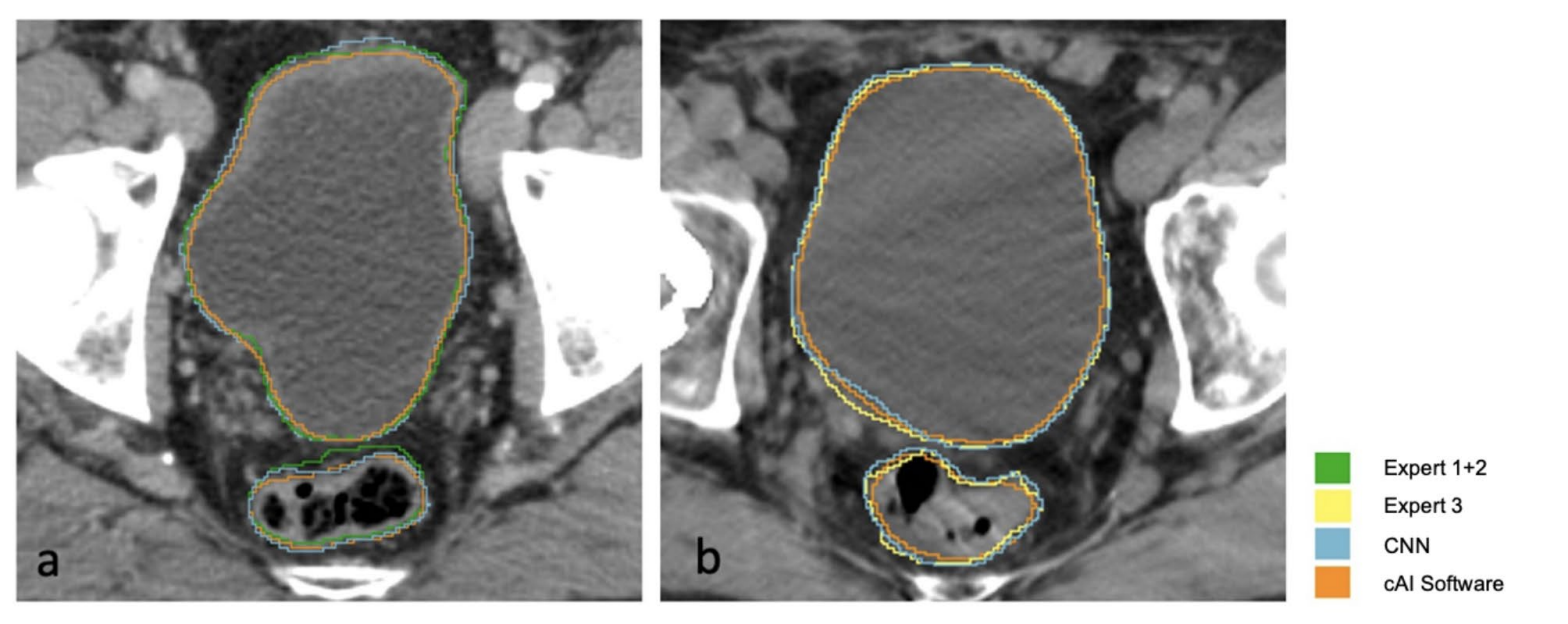
Representative CT datasets from the validation (**a**) and test cohorts (**b**). The resulting DSC values for these patients are the same as the median Dice-Sørensen Coefficient (DSC) for the cohorts. Segmentations: expert (green), independent expert (yellow), CNN (blue), cAI (orange).

In the test cohort, the CNN-segmentations were compared to those of the expert 3 (III), resulting in a median DSC_Bladder_ = 0.96 and DSC_Rectum_ = 0.88. Finally, comparison of cAI against independent expert contours, resulted in a median DSC bladder = 0.95 and rectum = 0.82. Significant differences were observed between CNN and cAI (validation bladder: p < 0.0001, rectum p < 0.0001; test bladder: p = 0.0115, rectum p = 0.0105; Wilcoxon matched-pairs signed rank test, two-tailed). Figure 2 shows the segmentations for both cohorts. For detailed results, refer to Table 2.

**Table 2:** Metrics of the analyzed validation and test cohort. The median Dice-Sørensen Coefficient (DSC) and interquartile range are shown. Results are listed for each organ at risk individually.

Cohort 1: Validation cohort (N = 29) Datasets from Freiburg, Germany (Center 1) and Nanjing, China (Center 2)		
DSC	I: CNN vs. Expert 1 + 2	II: cAI Software vs. Expert 1 + 2
Bladder	**0.93** (0.92–0.94)	**0.90** (0.84–0.94)
Rectum	**0.87** (0.83–0.89)	**0.78** (0.72–0.81)

Cohort 2: Test cohort (N = 16) Datasets from Limassol, Cyprus (Center 3)		
DSC	III: CNN vs. Expert 3	IV: cAI Software vs. Expert 3
Bladder	**0.96** (0.96–0.97)	**0.95** (0.94–0.96)
Rectum	**0.88** (0.85–0.90)	**0.82** (0.76–0.85)

#### Visual analysis

**Fig. 3:**
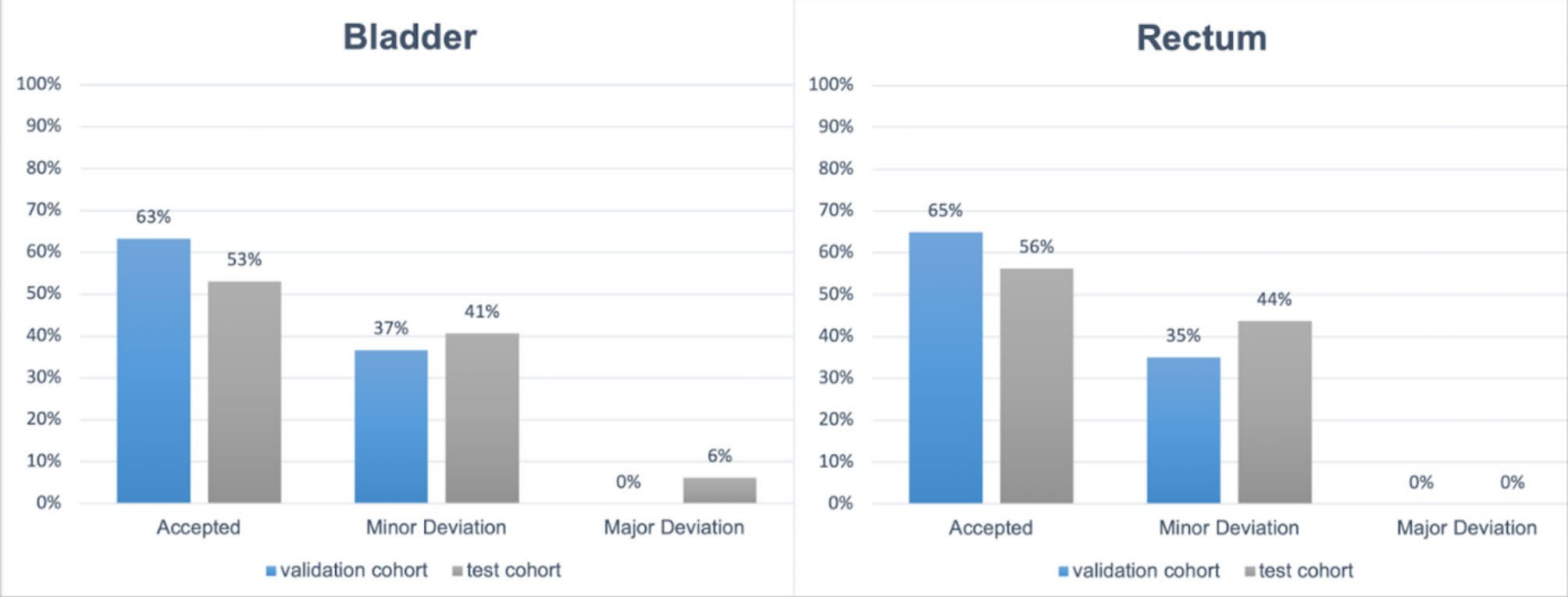
Column chart of the visual analysis for both organs at risk, bladder (on the left) and rectum (on the right). The results for the validation cohort are displayed in the blue column, the test cohort in grey.

No structure showed a gross deviation from the organ boundaries and was classified as a 'Major Deviation'. Similar classifications were made for the test cohort from center 3, except for one bladder segmentation, which was classified as ‘Major Deviation’. The percentage distribution of both experts is shown in Fig. 3.

#### Turing test

The evaluation of the Turing test calculated a misclassification rate of 47.5% for bladder and 49.2% for rectum contours in the validation cohort. Thus, the threshold of 30% has been surpassed. Regarding the test cohort of center 3, the observers classified 43.8% of the bladder and 39.1% of the rectum segmentations wrong, exceeding the threshold. Detailed results are shown in Table 3 (for further information please check the Supplementary, Table S2).

**Table 3:** Turing Test results of the validation cohort (above) and the test cohort (below). Results are listed for each organ at risk (OAR). Additionally, the mean results for CNN and expert segmentations and the total misclassification rate are displayed.

Cohort 1: Validation cohort (N = 29) Datasets from Freiburg, Germany (center 1) and Nanjing, China (center 2)				
Misclassification rate	Bladder		Rectum	
	CNN	Expert	CNN	Expert
Mean	40.0%	55.0%	46.7%	51.7%
Total	**47.5%**		**49.2%**	

Cohort 2: Test Cohort (N = 16) Datasets from Limassol, Cyprus (center 3)				
Misclassification rate	Bladder		Rectum	
	CNN	Expert	CNN	Expert
Mean	34.4%	40.6%	31.3%	46.9%
Total	**43.8%**		**39.1%**	

## Discussion

CNNs have become an important tool for automizing tasks in radiotherapy over the last years and therefore, are part in radiation treatment planning^12,13^. Various aspects of medical image processing can be optimized and supported by AI software, such as in radiation planning or adaptive planning^14^. This study shows the possibility and evaluation of bladder and rectum segmentations with a freely available CNN that was trained from scratch on multicentric PSMA-PET/CT data.

The CNN demonstrated good and reliable performance, achieving a median DSC_Bladder_ = 0.93 (0.92–0.94) and DSC_Rectum_ = 0.87 (0.83–0.89) in the quantitative analysis of the validation cohort with a good qualitative and clinically usable evaluation. To our knowledge, there are few similar studies that train and evaluate CNNs on CT datasets with a combination of quantitative and qualitative assessment of auto-segmentations for prostate cancer radiation treatment planning.

For qualitative evaluation, a visual analysis of the segmentation quality can be done by classifying them by an expert. This evaluation can be performed in various ways: It can be classified into three categories, as in Sherer et al.^9^, or into four categories (e.g., acceptable/small adaptation/big adaptation/not acceptable), as seen in Savenije et al.^15^ and Nachbar et al.^16^. Künzel et al.^17^ characterized the quality with a 4-level Likert-scale. This assessment is important to evaluate the usability and the potential for clinical implementation. Another possibility is to apply the Turing test on the CNN segmentations. As part of this, the observers were unable to differentiate between human and CNN-generated segmentations in this project. The high misclassification rate of > 40% demonstrates that automatically generated segmentations can imitate experienced clinicians and may therefore support radiation treatment planning workflow. To summarize, the visual assessment and the results of the Turing test supports the reliability and clinical applicability of the trained CNN. To improve reliability, future research projects should include more observers, preferably from different institutes as performed by Gooding et al.^18^.

However, other CNN projects similar to ours used planning-CT or MRI datasets for OAR segmentation in PCa RT planning, rather than only diagnostic CT datasets of PET/CTs. One study using the same CNN, conducted by Lorenzen et al.^19^, reached a DSC_Bladder_ = 0.98 (0.96–0.99) and DSC_Rectum_ = 0.97 (0.96–0.98) by training and testing MRI-datasets. Other studies such as Rhee et al.^20^ evaluated a self-trained CNN-based auto-contouring tool for target volume and OAR definition on female pelvis CTs with cervical cancer undergoing RT. The training was done from scratch by four physicians of their clinic. Results for bladder and rectum segmentation were a mean DSC_Bladder_ = 0.89 ± 0.09 and DSC_Rectum_ 0.81 ± 0.09. Furthermore, Sartor et al.^21^ trained a cloud-based CNN by Trägårdh et al.^22^ with manually created OAR structures by a clinical experienced expert, validated and tested on randomized CT datasets from PET/CT scans from anorectal and cervical cancer patients. As an assessment, they calculated the DSCs and categorized the structures into four categories. Their median DSC for the urinary bladder was 0.84 with 30% of their contours categorized as ‘not acceptable’. A rectum contour was not delineated. Therefore, the applied CNN in our study achieved results comparable or even better to those of former studies. Thus, our results confirm that it is possible to train the CNN using diagnostic CT datasets.

The good performance of our CNN on planning CTs in the validation cohort (DSC_Bladder_ > 0.96 and DSC_Rectum_ > 0.85) showed that a transfer to another modality with the same radiographic technique but different patient preparation such as bladder and rectum filling (PET/CT to planning-CT) is possible. This is in line with results of an in-house project, which showed that uni-modality trained CNNs are able to process conebeam CTs and megavoltage CTs^23^. The intermodal applicability is of particular interest for offline-adaptive planning workflows, who aim for a planning optimization based on the individual filling conditions of bladder and rectum in pelvic irradiation.

In contrast to other projects, previously untrained personnel conducted the training of the CNN whilst usually experienced experts are involved. The performed primary check of the student's reference structures eliminated systematic errors and supervision ensured a correct training. Thus, our results demonstrate, that it is possible for untrained personnel to acquire the necessary skills in a short time. This is a significant benefit for hospitals seeking to incorporate deep learning support, without major personnel and financial expense.

In addition to the personnel and financial advantages, our approach enables to train CNNs locally, allowing to adapt to local conditions, such as diagnostic equipment, individual positioning aids or individual preferences. This concept might reach superior performances in contrast to commercial AI software, which is centrally trained and optimized by experienced employees to enable globally applicable organ segmentation. The used cAI provided by Limbus Contours is one of the most commonly used AI software for segmentation tasks and has been part of various studies showing its impact on planning workflow and clinical usability^24,25^. Although the cAI reached very good results with median DSCs > 0.8, our CNN outperformed the cAI, supporting the hypothesis of improved results by locally trained CNNs. Additionally, the cAI did not generate segmentations in one dataset with coarse pixels, wheras the locally trained CNN performed well (DSC_Bladder_ = 0.92, DSC_Rectum_ = 0.81). To put the results in context to other freely available segmentation programs, a further quantitative and qualitative comparison with the Total Segmentator (TS) by Wasserthal et al.^6^ was conducted on the validation cohort (additional data not shown). Bladder segmentations were created, and the DSC was calculated on 28 patients (median DSC = 0.88 (IQR: 0.86–0.91)). No segmentation was considered as ‘Accepted’ for radiation treatment planning in the visual analysis, 50% were classified as ‘Minor Deviation’ and 50% as ‘Major Deviation’. In 22 out of 28 datasets, parts of the prostate were delineated as parts of the bladder and therefore did not respect the organ boundaries. In summary, the quality of the TS segmentations is not good enough for RT planning.

We acknowledge the following limitations of our study. Our study includes only a limited number of datasets for training and testing. Unfortunately, there is no standardized number of datasets for CNN training in the literature. A review by Samarasinghe et al.^26^ revealed a wide range of included datasets for pelvic segmentation. Feng et al.^27^ included the smallest number of patients, with 40 patients (30 training/10 test), while Liu et al.^28^ provided the largest project with 1104 (771 training/193 validation/140 test). Therefore, our number of datasets is in the range of available studies. Once the optimal size of the training dataset will have been identified, data collecting may become easier, and an overfitting of neural network can be prevented.

Additionally, only two OARs, the bladder and the rectum, were analyzed in this study. However, they are the most important OARs for planning of pelvic irradiations due to possible toxicities. In future studies, segmentation of further OARs, such as the femoral heads, bowel loops, or penile bulb, may be added through transfer learning, enhancing the clinical usability of the CNN.

In summary, in this study, a CNN for automated segmentation of the OARs bladder and rectum was trained on PSMA PET/CT images and tested on PET/CT and planning CT images. The student-trained CNN yielded very good DSCs, generated clinically useful segmentations and outperformed a centrally trained commercial AI software. The CNN performed well on different CT modalities including diagnostic PET/CT scans and planning-CT scans. CNNs can be trained locally without great personal or financial expanses reaching a clinical meaningful benefit.

## Electronic supplementary material

Below is the link to the electronic supplementary material.

Supplementary Material 1

## Data Availability

The generated and analyzed datasets are available from the corresponding author upon reasonable request. The analyzed Convolutional Neural Network is an open source code from Isensee et al. (10.1038/s41592-020-01008-z).
