# Supplementary material for: A student trained convolutional neural network competing with a commercial AI software and experts in organ at risk segmentation
Source: Sci Rep. 2024 Oct 29;14:25929. doi: 10.1038/s41598-024-76288-y (PMC11522297; doi:10.1038/s41598-024-76288-y)
Supplement: Supplementary file 1 — Supplementary Material 1 [file 41598_2024_76288_MOESM1_ESM.docx]

# **A student trained Convolutional Neural Network competing with a commercial AI software and experts in organ at risk segmentation**

Sophia L. Bürkle *^a^, Dejan Kuhn ^b^, Tobias Fechter ^b,c^, Gianluca Radicioni ^a^, Nanna Hartong ^a^,
Martin T. Freitag^d^, Xuefeng Qiu^e^, Efstratios Karagiannis ^f^, Anca-Ligia Grosu ^a,c^, Dimos Baltas ^b,c^,
Constantinos Zamboglou ^a,c,f,g^, Simon K. B. Spohn ^a,c,g^

a Department of Radiation Oncology, University Medical Center Freiburg, Faculty of Medicine, University of Freiburg, Germany.

b Division of Medical Physics, Department of Radiation Oncology, University Medical Center Freiburg, Faculty of Medicine, University of Freiburg, Germany.

c German Cancer Consortium (DKTK), Partner Site Freiburg, Germany.

d Department of Nuclear Medicine, University Medical Center Freiburg, Faculty of Medicine, University of Freiburg, Germany.

e Department of Urology, Affiliated Drum Tower Hospital, Medical School of Nanjing University, China.

f German Oncology Center (GOC), European University of Cyprus, Limassol, Cyprus.

g Berta-Ottenstein-Programme, Faculty of Medicine, University of Freiburg, Germany.

* corresponding author:

Sophia L. Bürkle, [sophia.buerkle@uniklinik-freiburg.de](mailto:sophia.buerkle@uniklinik-freiburg.de)

Universitätsklinikum Freiburg, Department of Radiation Oncology

Robert-Koch-Straße 3, D-79106 Freiburg

**Supplementary**

|  | Center 1:  Freiburg, Germany | Center 2:  Nanjing,  China | Center 3:  Limassol, Cyprus |
| --- | --- | --- | --- |
| n _total_ | 122 | 50 | 16 |
| n _training_ | 99 | 43 | 0 |
| n _validation_ | 23 | 7 | 0 |
| n _test_ | 0 | 0 | 16 |
|  |  |  |  |
| age in years  (mean, SD) | 69.1  $\sigma$= 7.7 | 69.9  $\sigma$= 6.5 | 71.1  $\sigma$= 5.1 |
|  |  |  |  |
| iPSA in ng/ml (median, range) | 12.9  (8.1-20.5) | 29.81  (7.9-29.8) | 7.7  (6.76-11.56) |
|  |  |  |  |
| ISUP Score |  |  |  |
| 1 | 5 | 4 | 4 |
| 2 | 31 | 15 | 4 |
| 3 | 37 | 37 | 6 |
| 4 | 25 | 25 | 2 |
| 5 | 22 | 22 | 0 |
|  |  |  |  |
| T status |  |  |  |
| T1 | 1 | 0 | 5 |
| T2 | 57 | 16 | 9 |
| T3 | 50 | 34 | 2 |
| T4 | 0 | 0 | 0 |
|  |  |  |  |
| N status |  |  |  |
| N0 | 90 | 6 | 16 |
| N1 | 28 | 4 | 0 |
| Nx | 2 | 42 | 0 |
|  |  |  |  |
| M status |  |  |  |
| M0 | 110 | 50 | 15 |
| M1 | 5 | 0 | 0 |
| Mx | 5 | 0 | 1 |

Table S1: Patient characteristics. This table displays the cohort distribution and further information such as age, histopathologic features, prostate specific antigen (PSA) level, and TNM-classification.

| Cohort 1: validation cohort (n = 29)  Datasets from Freiburg, Germany (Center 1) and Nanjing, China (Center 2) | | | | | | | | | | | | | |  |  |
| --- | --- | --- | --- | --- | --- | --- | --- | --- | --- | --- | --- | --- | --- | --- | --- |
|  | | Bladder | |  | | | Rectum | | | | | |  |  |  |
|  | | ***CNN*** | ***Experts*** | | |  | | |  | | ***CNN*** | ***Experts*** | | | |
| *Observer* | | *Misclassification rate* | | | |  | | |  | | *Misclassification rate* | | | | |
| A | | 33.3% | 26.7% | | |  | | |  | | 20.0% | 60.0% | | | |
| B | | 60.0% | 86.0% | | |  | | |  | | 80.0% | 66.7% | | | |
| C | | 26.7% | 40.0% | | |  | | |  | | 26.7% | 40.0% | | | |
| D | | 40.0% | 66.7% | | |  | | |  | | 60.0% | 26.7% | | | |
| mean | | **40.0%** | **55.0%** | | |  | | |  | | **46.7%** | **51.7%** | | | |
| total | | **47.5%** | | | |  | | |  | | **49.2%** | | | | |
|  |  | | | |  | | |  | |  | | | | |  |
| Cohort 2: test cohort (n = 16)  Datasets from Limassol, Cyprus (Center 3) | | | | | | | | | | | | | |  |  |
|  | | Bladder | |  | | | Rectum | | | | | |  |  |  |
|  | | ***CNN*** | ***Experts*** | | |  | | |  | | ***CNN*** | ***Experts*** | | | |
| *Observer* | | *Misclassification rate* | | | |  | | |  | | *Misclassification rate* | | | | |
| A | | 37.5% | 25.0% | | |  | | |  | | 12.5% | 37.5% | | | |
| B | | 37.5% | 37.5% | | |  | | |  | | 12.5% | 37.5% | | | |
| C | | 25.0% | 37.5% | | |  | | |  | | 25.0% | 62.5% | | | |
| D | | 37.5% | 62.5% | | |  | | |  | | 75.0% | 50.0% | | | |
| mean | | **34.4%** | **40.6%** | | |  | | |  | | **31.3%** | **46.9%** | | | |
| total | | **43.8 %** | | | |  | | |  | | **39.1%** | | | | |

Table S2: Detailed results of the Turing Test are listed for the validation cohort and test cohort, separately for each organ at risk. This table shows the individual results of the four clinical radiation oncologists, referred to as observers A-D.
